# Cost-effectiveness of stereotactic large-core needle biopsy for nonpalpable breast lesions compared to open-breast biopsy

**DOI:** 10.1038/sj.bjc.6601520

**Published:** 2004-01-20

**Authors:** J H Groenewoud, R M Pijnappel, M E van den Akker-van Marle, E Birnie, T Buijs-van der Woude, W P Th M Mali, H J de Koning, E Buskens

**Affiliations:** 1 Department of Public Health, Erasmus MC, University Medical Center Rotterdam, PO Box 1738, 3000 DR Rotterdam, The Netherlands; 2 Department of Radiology, Martini Ziekenhuis Groningen, PO Box 30033, 9700 RM Groningen, The Netherlands; 3 Julius Center for Health Sciences and Primary Care, University Medical Center Utrecht, PO Box 85500, 3508 GA Utrecht, The Netherlands; 4 Department of Radiology, University Medical Center Utrecht, PO Box 85500, 3508 GA Utrecht, The Netherlands

**Keywords:** breast radiography, biopsies, cost-effectiveness, cancer screening

## Abstract

This paper demonstrates that the introduction of large-core needle biopsy (LCNB) replacing needle-localised breast biopsy (NLBB) for nonpalpable (screen-detected) breast lesions could result in substantial cost savings at the expense of a possible slight increase in breast cancer mortality. The cost-effectiveness of LCNB and NLBB was estimated using a microsimulation model. The sensitivity of LCNB (0.97) and resource use and costs of LCNB and NLBB were derived from a multicentre consecutive cohort study among 973 women who consented in getting LCNB and NLBB, if LCNB was negative. Sensitivity analyses were performed. Replacing NLBB with LCNB would result in approximately six more breast cancer deaths per year (in a target population of 2.1 million women), or in 1000 extra life-years lost from breast cancer (effect over 100 years). The total costs of management of breast cancer (3% discounted) are estimated at £4676 million with NLBB; introducing LCNB would save £13 million. The incremental cost-effectiveness ratio of continued NLBB *vs* LCNB would be £12 482 per additional life-year gained (3% discounted); incremental costs range from £-21 687 (low threshold for breast biopsy) to £74 378 (high sensitivity of LCNB).

In Western societies, breast cancer is the most common malignancy in women. This disease and the ensuing mortality are considered the major public health problems. Mass screening for breast cancer is deemed effective in reducing breast cancer mortality. Studies have shown that early detection of breast cancer may reduce breast cancer mortality (Nystrom *et al*, 2002; Otto *et al*, 2003). As a result of screening, nonpalpable small breast lesions are increasingly detected.

Open-breast biopsy has long been considered the gold standard to determine malignancy in palpable and nonpalpable lesions. Despite excellent test characteristics (Verkooijen *et al*, 2000), open-breast biopsy has disadvantages. The surgical procedure may cause anxiety, morbidity or even mortality, and may leave women with a permanent deformation and scar of the breast. Women may need to be hospitalised and high-cost surgical and anaesthetic resources are used. In search of clinically equivalent less invasive diagnostic alternatives, stereotactic large-core needle biopsy (LCNB) has been introduced, particularly for nonpalpable lesions (Gisvold *et al*, 1994; Britton *et al*, 1997; Pijnappel *et al*, 1997; Jackman *et al*, 1999).

A decision analytic approach based on data obtained from literature suggested that core biopsy and open-breast biopsy may be clinically equivalent, core biopsy being less costly than open-breast biopsy (Hrung *et al*, 1999). Prior to our study, actual direct empirical comparison of the diagnostic procedures had not been performed. In a multicentre clinical study, both techniques were compared (Verkooijen, 2000). Using a validated microsimulation model, we set out to assess the effect of stereotactic LCNB replacing conventional needle-localised breast biopsy (NLBB) as diagnostic procedure in women with nonpalpable screen-detected breast lesions in terms of breast cancer mortality, life-years and cost-effectiveness.

## MATERIALS AND METHODS

### Clinical study – the COBRA study

The test characteristics of stereotactic LCNB (14-gauge) were obtained from a prospective multicentre consecutive cohort study (the COBRA study) (Verkooijen, 2002). This study was performed between April 1997 and February 2000 to assess the diagnostic accuracy of LCNB, as compared to NLBB in women with nonpalpable breast lesions. After having given informed consent to participate in the study, the women underwent core biopsy first. Biopsies were done by specially trained radiologists; at least five biopsy specimens were obtained from each lesion. After the core biopsy, all women underwent open-breast biopsy as well (gold standard); they underwent therapeutic surgery when the LCNB yielded breast cancer or NLBB in cases of LCNB without malignancy. In total, 973 consecutive women (1029 lesions) were enrolled. In 813 women (858 lesions), the LCNB procedure could be completed successfully. Three possible outcomes of LCNB were distinguished: (1) malignancy; (2) benign lesion; (3) inconclusive result (normal tissue, high-risk lesion). In the case of an inconclusive result, additional NLBB has been recommended (Verkooijen, 2002). In our study, the sensitivity of LCNB was calculated including this second-step diagnostic procedure for inconclusive results.

### Structure of the microsimulation model

To study the impact of LCNB and NLBB on health effects and costs, we used the simulation model MISCAN (van Oortmarssen *et al*, 1990; Boer *et al*, 1995; de Koning *et al*, 1995). The microsimulation screening analysis program was originally designed to evaluate the introduction of mass screening for breast cancer and to support related decision-making (de Koning *et al*, 1991; van den Akker-van Marle *et al*, 1997; Boer *et al*, 1998). The model is described in more detail in Appendix A. In summary, the core of the model consists of ‘simulated life histories’ including the natural history of breast cancer. The natural history is modelled as the onset of breast cancer, the transition through four possible preclinical invasive stages with increasing tumour diameters (T1a, T1b, T1c and T2+) and the transition from one of the preclinical stages into a clinically diagnosed stage (DCIS, T1a, T1b, T1c or T2+) until the women die from breast cancer or from other causes. Breast cancer screening is introduced as an intervention in the natural history, possibly resulting in the detection of breast cancer in earlier (preclinical, presymptomatic) stages.

The output of the model yields a number of estimates of the effect of screening and subsequent outcome. The numbers of women invited and screened are assessed, as well as the resulting number of true-positive and false-positive test results, and the number of cancers diagnosed outside the screening programme. The output also includes the numbers of diagnostic procedures for breast tumours detected by screening and for breast cancers detected outside the context of the breast cancer-screening programme, and the number of breast cancer therapies. Moreover, the model calculates the number of breast cancer deaths and life-years lost due to cancer.

Cost-effectiveness was calculated by adding a profile of costs over time to each disease state.

### Model assumptions

#### Screening programme

In the model, we assumed a breast cancer-screening programme lasting 27 years. This programme implies biennial screening mammography for women aged 50–75 years, in accordance with the current screening policy in the Netherlands (current target population: 2.1 million women). Health effects were considered for the remaining life expectancy of the cohort simulated (maximum age 100 years).

#### Diagnostic scenarios

**Figure 1:**
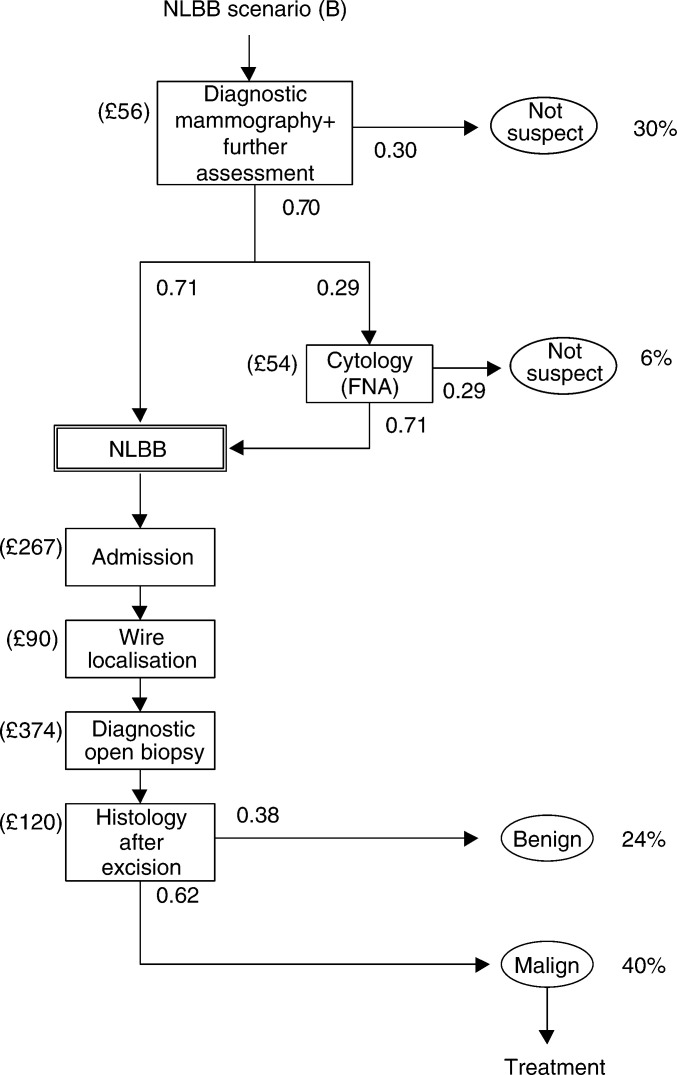

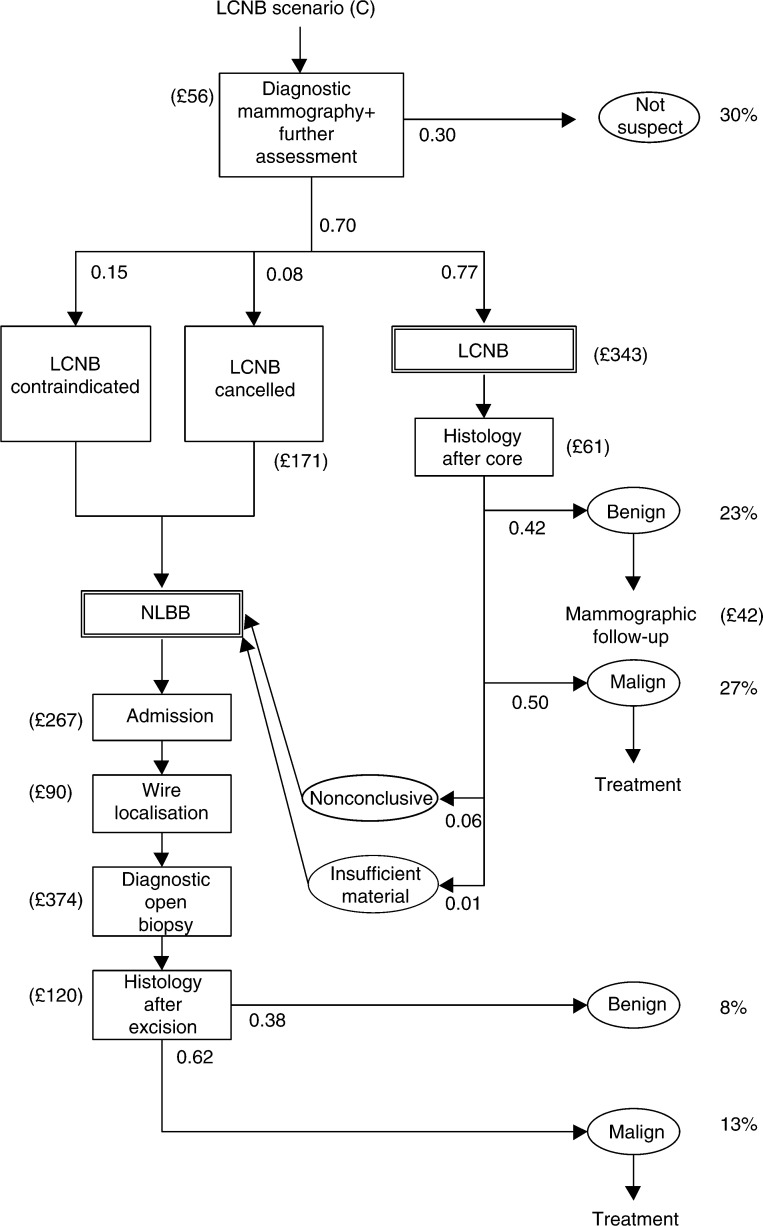
Diagnostic procedures for nonpalpable breast lesions detected at screening (model input). Needle-localised breast biopsy (scenario (B)) and LCNB (scenario (C)), FNA=fine needle aspiration (cytology), malign: invasive breast carcinoma or ductal carcinoma *in situ*, nonconclusive: normal tissue or high-risk lesion.

We simulated three scenarios: (A) the situation without mass screening; (B) mass screening with NLBB as diagnostic work-up for palpable and nonpalpable breast lesions; and (C) mass screening with LCNB replacing NLBB as diagnostic work-up for nonpalpable lesions, followed by NLBB if the core biopsy indicates normal breast tissue or a high-risk lesion (Verkooijen, 2000). We used the data from our empirical study to quantify the relevant stages in the diagnostic scenarios. Figure 1

shows the diagnostic strategies (B) and (C) for nonpalpable screen-detected breast lesions. In scenario (B), after a positive screen examination, women with nonpalpable lesions will be scheduled for NLBB, unless diagnostic mammography or fine needle aspiration (FNA) cytology indicate no malignancy (‘not suspect’). Preoperative FNA cytology precedes about one-third of NLBB procedures in nonpalpable breast lesions. In scenario (C), women with a positive screen examination are scheduled for LCNB, unless diagnostic mammography indicates the absence of malignancy (‘not suspect’). Preceding LCNB, no FNA cytology is assumed. Women in scenario (C) will undergo NLBB if LCNB is contraindicated, if LCNB is cancelled (for reasons including too small breasts for adequate compression, negative stroke margins, patients not enduring prone position or lesions being localised too close to the chest wall), if the tissue samples obtained by LCNB are nonrepresentative, or if tissue samples indicate normal breast tissue or a ‘high-risk’ lesion. Among the contraindications for LCNB are coagulopathy, anticoagulant use and the inability to maintain prone position for 1 h. If invasive cancer or DCIS is diagnosed, women undergo definite treatment, that is, breast-conserving therapy or mastectomy, or surgical excision, respectively. Women with benign lesions are offered follow-up mammography. Women with screen-detected breast lesions that are palpable and women with breast lesions detected outside the screening programme are considered to undergo open-breast biopsy (not shown in Figure 1).

#### Test characteristics

NLBB was considered to be the gold standard diagnostic procedure with 100% sensitivity. The test characteristics of LCNB were obtained from the COBRA study.

### Costs

#### Diagnostic phase

The costs of the diagnostic phase were calculated as the resource use in the diagnostic stage multiplied with cost-per-unit estimates. The relevant resource items were LCNB, NLBB, histopathological examination, hospitalisation, day care, general practitioner (GP), diagnostic mammography, other diagnostic procedures (e.g. ultrasound and MR imaging), FNA and outpatient visit. The actual economic costs per unit of LCNB, NLBB and histopathological examination were obtained from five participating Dutch hospitals. Due to practice variation, the costs of hospitalisation for diagnostic excision biopsy were averaged for treatment setting (day care for about half of the biopsies, and hospitalisation for the other half with a mean hospital stay of 3 days) (Buijs-van der Woude *et al*, 2001). The costs per unit of resource use were calculated as actual economic costs, that is, including medical staff, other personnel, disposables, fixed equipment and a mark-up for fixed costs and overheads (Finkler, 1982). The costs of GP care and outpatient visits were based on Dutch reference guidelines (Oostenbrink *et al*, 2000).

#### Treatment phase

The costs of breast cancer treatment were obtained from previous studies (de Koning *et al*, 1991). Reimbursement fees were used to estimate the costs of primary and adjuvant therapies. The costs of palliative care were obtained from studying the records of patients with breast cancer metastases.

Costs were originally calculated in Dutch currency and converted into UK£ using the 2002 purchasing-power parity (PPP: DFL 1.00=UK£0.312). To take account of time preference, the costs estimates over time were adjusted with 3% (baseline) and 5% (sensitivity analysis) annual discount rate. To compute the cost-effectiveness, equal discount rates were applied to the effects as well (Lipscomb *et al*, 1996). Costs and references are summarised in Table 1

**Table 1:** Model parameters and range of sensitivity analyses

**Parameter**	**Base case**	**Sensitivity analyses**	**Reference or source**
*Test performance*			
Sensitivity of NLBB	1.00		Assumed
Sensitivity of LCNB^a^	0.97	0.96/0.99^b^	COBRA study
			
*Probability of nonmalignant diagnosis of a nonpalpable lesion after*			
Diagnostic mammography/further assessment	0.30	0.15/0.00	Dutch BCSP
Cytology (FNA)	0.25		Dutch BCSP
NLBB	0.38		Dutch BCSP
LCNB	0.50		(Verkooijen, 2000)
Preoperative FNA for nonpalpable lesions (scenario (B))	0.29		(Pijnappel *et al*, 2001)
Contraindication for LCNB (scenario (C))	0.15		COBRA study
Unsuccessful LCNB (cancelled or nonrepresentative material) (scenario (C))	0.08		COBRA study
Proportion of nonpalpable suspicious breast lesions at screening by mammography	±60%^c^	±75%^d^	Assumed/(BreastScreen Victoria, 2000)
			
*Costs*			
Diagnostic stage			
General practitioner	£12		(Oostenbrink *et al*, 2000)
Diagnostic mammogram	£56		(de Koning *et al*, 1991)
MR imaging	£264		(de Koning *et al*, 1991)
Ultrasound (additional costs only)	£9		(de Koning *et al*, 1991)
FNA	£54		(de Koning *et al*, 1991)
Outpatient visit	£30		(Oostenbrink *et al*, 2000)
LCNB	£343	£189–611	(Buijs-van der Woude *et al*, 2001)
NLBB	£374		(Buijs-van der Woude *et al*, 2001)
Admission or hospitalisation	£267^e^		(Buijs-van der Woude *et al*, 2001)
Wire localisation	£90		(Buijs-van der Woude *et al*, 2001)
Histopathological examination			
LCNB	£61		(Buijs-van der Woude *et al*, 2001)
NLBB	£120		(Buijs-van der Woude *et al*, 2001)
Follow-up in disease-free stage (per year)			
First disease-free year	£125		(de Koning *et al*, 1991)
Every following year	£88		(de Koning *et al*, 1991)
Primary therapy			
BCT, without booster (invasive tumours)	£4046		(de Koning *et al*, 1991)
BCT, internal booster (invasive tumours)	£5558		(de Koning *et al*, 1991)
BCT, external booster (invasive tumours)	£4774		(de Koning *et al*, 1991)
Mastectomy followed by radiotherapy (invasive tumours)	£3638		(de Koning *et al*, 1991)
Mastectomy without radiotherapy (invasive tumours)	£2091		(de Koning *et al*, 1991)
Excision followed by radiotherapy (DCIS)	£2563		(de Koning *et al*, 1991)
Excision without radiotherapy (DCIS)	£310		(de Koning *et al*, 1991)
Amputation (DCIS)	£1749		(de Koning *et al*, 1991)
Adjuvant therapy			
Chemotherapy	£1020		(de Koning *et al*, 1991)
Hormonal therapy	£561		(de Koning *et al*, 1991)
Palliative treatment			
M0 (no metastasis)	£12 158		(de Koning *et al*, 1991)
M1 (metastatic disease)	£12 158		(de Koning *et al*, 1991)

BCSP=breast cancer-screening programme; BCT=breast-conserving therapy.

a LCNB, including additional open-breast biopsy in the case of normal tissue or high-risk lesion.

b Lower and upper limit of the 95% confidence interval of the sensitivity. The sensitivity of LCNB for DCIS is 0.94 (95% CI 0.90–0.97), for invasive carcinoma 0.99 (0.99–1.00).

c Tumour stages DCIS, T1a and T1b are considered to involve nonpalpable breast tumours.

d Nonpalpable and palpable breast tumours are distributed over all tumour stages: as the size of the tumours increased, a smaller proportion was nonpalpable(2000).

e The mean cost of hospitalisation for all NLBBs; half of such biopsies were performed in day care; in the other half of cases, hospitalisation was necessary (the mean hospital stay for all open breast biopsies was 3 days) (Buijs-van der Woude *et al*, 2001).

.

### Sensitivity analysis

Sensitivity analysis was performed to quantify the impact of variations in essential model parameters and assumptions on cost-effectiveness (Table 1).

First, the sensitivity of LCNB for DCIS and invasive breast lesions was varied using the limits of the 95% confidence intervals.

Second, the costs of LCNB were varied: the low and high LCNB costs correspond to the case where LCNB is employed highly concentrated in 10 of all Dutch hospitals or decentralised (in all 114 Dutch hospitals), respectively (Buijs-van der Woude *et al*, 2001).

Third, the costs in the NLBB scenario were varied, assuming that all NLBBs were done in day care, taking into account that there will be complications that require hospitalisation in 4% (mean costs of admission: £137).

Fourth, we simulated LCNB scenarios where 100 or 85% of referred women, respectively, would have further histopathologic assessment after clinical mammography and physical examination.

Finally, we varied the distribution of nonpalpable lesions at screening. In the basic model, all DCIS and lesions <1 cm in diameter (i.e. T1a and T1b) were considered nonpalpable, whereas T1c and T2+ lesions were regarded palpable. As a high estimate, we considered 75% of all lesions to be nonpalpable, with the proportion of nonpalpable tumours gradually decreasing with tumour size (BreastScreen Victoria, 2000).

## RESULTS

### Sensitivity of LCNB

Of the nonpalpable breast lesions, 58% (494 of 858) were malignant according to the gold standard: 20% (172 of 858) involved ductal carcinoma *in situ* (DCIS) and 38% (322 of 858) invasive carcinoma. With the LCNB procedure, 481 of 494 malignancies were detected: malignancy being the initial diagnosis at core biopsy (470 cases) or the diagnosis at surgery following a nonconclusive diagnosis (normal tissue or ‘high-risk’ lesion) at core biopsy (11 cases). Thus, the overall sensitivity of the LCNB procedure was 0.97 (95% CI: 0.96–0.99). The sensitivity for DCIS was 0.94 (161 of 172) (95% CI: 0.90–0.97) and for invasive breast cancer 0.99 (320 of 322) (95% CI: 0.99–1.00).

### Diagnostic phase

Table 2

**Table 2:** Number of biopsies and breast cancers diagnosed with NLBB and LCNB^a^ as diagnostic procedure for breast lesions detected at screening^b^ (0% discounted)

	**NLBB scenario (B)**	**LCNB scenario (C)**
*Within the context of screening programme*		
No. of women undergoing biopsy (× 1000)	156.4	165.1
LCNB (nonpalpable lesions only)	0	73.1^c^
NLBB	156.4	92.0
Palpable lesions	61.9	62.1
Nonpalpable lesions	94.6	29.9^d^
No. of screen-detected cancers (× 1000)	116.5	115.8
LCNB (nonpalpable lesions only)	0	39.6
NLBB	116.5	76.2
Palpable lesions	57.4	57.7
Nonpalpable lesions	59.0	18.6
		
*Outside screening programme*		
No. of women undergoing biopsy (NLBB only) (× 1000)	1434.3	1435.2
Palpable lesions	1231.7	1232.3
Nonpalpable lesions	202.6	202.9
No. of breast cancers diagnosed (× 1000)	789.3	789.8
Palpable lesions	706.1	706.4
Nonpalpable lesions	83.2	83.4

a Including additional NLBB for benign or high-risk lesions at LCNB.

b The screening programme consists of biennial screening mammography for women aged 50–75 years, and is carried out during a period of 27 years.

c In an additional 6200 women with nonpalpable lesions, the histological diagnosis of the core biopsy was normal tissue or high-risk lesion; these women therefore undergo NLBB.

d Of 29 900 women with nonpalpable lesions, 6200 underwent LCNB first, which led to the diagnosis of normal tissue or high-risk lesion.

shows the numbers of diagnostic procedures for palpable and nonpalpable lesions in the scenarios (B) (NLBB) and (C) (LCNB) (model calculations). In the NLBB scenario, 156 400 women (61 000 with palpable lesions and 94 600 with nonpalpable lesions) undergo NLBB; this is 64% of all women referred because of a positive screen examination. In the LCNB scenario, 165 100 referred women (68%) undergo biopsy: 62 100 have palpable lesions and undergo NLBB, 103 000 women have nonpalpable lesions and are scheduled for LCNB. Of the 103 000 women with nonpalpable lesions, 29 900 finally undergo NLBB.

In the NLBB scenario, 116 500 cancers are detected at screening (4.8 per 1000 women screened): 16 400 (14.1%) *in situ* cancers (DCIS), 42 700 (36.6%) T1a or T1b tumours, and 57 400 (49.3%) T1c or T2+ tumours. In the LCNB scenario, 115 800 cancers are detected at screening (4.7 per 1000 women screened). The tumour size distribution is somewhat less favourable, with 15 700 (13.5%) *in situ* tumours, 42 500 (36.7%) T1a or T1b tumours, and 56 700 (49.8%) T1c or T2+ tumours.

### Effects and costs

Table 3

**Table 3:** Mortality effects, cost and cost-effectiveness of three scenarios: no breast screening, screening with NLBB as diagnostic work-up and screening with LCNB as diagnostic work-up

			**Scenario (B) (NLBB)**		**Scenario (C) (LCNB)**	
	**Scenario (A) (no screening)**		**(difference with no screening)**			
Discount rate	0%	3%	0%	3%	0%	3%
*Effectiveness*						
Deaths from breast cancer	351 364	140 520	−31 195	−16 180	−31 029	−16 099
*Per year of screening*			*−1155*		*−1149*	
Life-years lost from breast cancer (× 1000)	6374	2395	−513.9	−202.6	−511.2	−201.6
						
*Costs (× 10*^*6*^* £)*						
Screening	0	0	+635	+432	+635	+432
Diagnostics	1802	910	−70	−40	−93	−55
Primary treatment	3059	1296	+87	+81	+91	+83
Follow-up	1136	454	+56	+30	+55	+29
Palliative care	4272	1708	−379	−197	−377	−196
Total	10 269	4369	+328	+307	+310	+294
						
*Cost-effectiveness*^a^ *(£)*						
Costs per life-year gained	—	—	639	1515	606	1459

a To calculate the cost-effectiveness, the difference in costs between the situation without mass screening (scenario (A)) and that with mass screening (scenarios (B) and (C), respectively) is divided by the difference in effects.

displays the estimated impact on breast cancer mortality and (quality-adjusted) life-years. Without screening, 351 364 women would die of breast cancer (effect over 100 years, 0% discounted). In scenario (B) (NLBB), breast cancer mortality would be reduced by 31 195 deaths (8.9%), that is, saving 514 000 life-years. Screening with LCNB as diagnostic work-up for nonpalpable lesions (scenario (C)) would prevent 31 029 breast cancer deaths (8.8%), thereby saving 511 000 life-years. If LCNB were to be implemented and replace NLBB, an extra six women would die of breast cancer for each year of screening.

Without screening, the total cost of the management of breast cancer would be £4369 million (3% discounted) for the next 100 years, of which £910 million (21%) would be spent on diagnostics. If screening is introduced, the total costs would increase by £307 million (7.0%) with NLBB as diagnostic work-up and by £294 million (6.7%) if LCNB is applied. Thus, the net (discounted) cost reduction of LCNB compared with NLBB would be £13 million, which can be mainly attributed to a reduction in diagnostic costs.

### Cost-effectiveness and sensitivity analysis

The costs per life-year gained resulting from screening would be £1515 with NLBB and £1459 for screening with LCNB (both costs and effects 3% discounted). The incremental cost-effectiveness ratio of NLBB *vs* LCNB is about £12 482 per life-year gained, that is extra costs of £12.70 million for NLBB compared to LCNB for 1017 additional life-years gained by NLBB.

Table 4

**Table 4:** Cost-effectiveness of LCNB and incremental cost-effectiveness ratio of NLBB *vs* LCNB (3% discounted); sensitivity analyses

**Parameter varied**	**Value**	**Cost per life-year gained; LCNB *vs* no mass screening^a^ (£)**	**ICER of NLBB *vs* LCNB^b^ (£)**
None (baseline variant)^c^		1459	12 482
Discount rate	0%	606	6891
	5%	2097	17 588
Sensitivity of LCNB	0.96	1469	6618
Sensitivity of LCNB (high)	0.99	1450	74 378
Costs of LCNB (low)	£189	1414	21 430
Costs of LCNB (high)	£611	1538	−3077
Costs of admission for NLBB (day care, 4% complications that require hospitalisation)	£137	1449	6326
Threshold for breast biopsy in the LCNB scenario (15% threshold)	0.15	1546	−4705
Threshold for breast biopsy in the LCNB scenario (0% threshold)	0.00	1632	−21 687
Threshold for breast biopsy in the LCNB scenario (15% threshold); costs of LCNB (low)	0.15/£189	1485	7297
Proportion of nonpalpable suspicious breast lesions at screening by mammography	±75%	1496	4625

a he cost per life-year gained is calculated through division of the difference in costs between the situation without mass screening (scenario (A)) and that with mass screening (scenario (C)) by the difference in effects.

b CER=incremental cost-effectiveness ratio. The ICER is calculated by dividing the difference in total cost of NLBB and LCNB by the difference in life years gained.

c Assumptions for the baseline variant are: 3% discount rate; sensitivity of LCNB 0.97; cost of LCNB £343; threshold for breast biopsy 30%; proportion of nonpalpable tumours (all DCIS and tumours in stages Ia and Ib) about 60%.

shows the results of the sensitivity analyses. At 5% discount rate, the incremental cost-effectiveness ratio rises to £17 588 (extra costs of £10.13 million for NLBB compared to LCNB to gain 576 additional life-years, both costs and effects 5% discounted). With sensitivity of LCNB of 0.99, the incremental cost-effectiveness ratio of NLBB *vs* LCNB was the highest (£74 378 per extra life-year gained, both costs and effects 3% discounted). The low cost of LCNB resulted in an incremental cost-effectiveness ratio of £21 430. The high cost of LCNB and lower thresholds for breast biopsy resulted in negative incremental cost-effectiveness ratios (lower costs of NLBB compared to LCNB, and no additional life-years gained).

## DISCUSSION

The current study shows that LCNB may be considered a cost-effective alternative to NLBB, that is, considerable cost savings are achieved at probably acceptable losses in terms of life-years. We estimated that, with the introduction of LCNB, costs of diagnosis and (primary) treatment of breast cancer would decrease by about £500 000 per year. Continued use of NLBB, on the other hand, would save more life-years. The pertaining incremental cost-effectiveness ratio of continued NLBB *vs* LCNB, that is, about £12 500 per life-year saved, may be ‘borderline’ in political decision-making about health-care services in the Netherlands, and will be well within the bounds of acceptable cost-effectiveness in many other countries. Further evaluation of quality of life in patients who underwent LCNB *vs* NLBB might be undertaken for a more elaborate comparison of particularly the long-term effects of LCNB *vs* NLBB in terms of quality-adjusted life-years.

Sensitivity analyses show that the incremental cost-effectiveness ratio of NLBB *vs* LCNB is most sensitive to relatively small variations in the sensitivity of LCNB. We considered NLBB as the gold standard diagnostic procedure, assuming 100% sensitivity. A recent study into the diagnostic accuracy of open-breast biopsy, however, showed that the sensitivity of open-breast biopsy dropped to 0.99 after 2 years of follow-up and to 0.96 after 5 years of follow-up, when taking into account the so-called interval lesions (Verkooijen *et al*, 2000). If the sensitivity of core biopsy and the sensitivity of open-breast biopsy indeed prove to be equal, then LCNB would dominate open-breast biopsy for all outcomes, that is, being the strategy with less morbidity and lower costs. Similarly, future developments of minimal invasive diagnostic techniques, such as vacuum-assisted biopsy, will probably have no further favourable effect on the cost-effectiveness, as long as the sensitivity of such technique is comparable to that of LCNB and costs are higher. However, for breast lesions consisting of microcalcifications only, vacuum-assisted biopsy might become a relevant alternative because the diagnostic accuracy of LCNB is significantly lower for such lesions than for other mammographic lesions (Pijnappel, 2002).

The cost of LCNB is another important factor for the cost-effectiveness. Centralisation of stereotactic equipment for LCNB, which implies lower costs per procedure, would be advisable from an economic perspective (Buijs-van der Woude *et al*, 2001). In case the costs of LCNB would considerably increase as compared to the base case situation, this might at the extreme result in a dominance of NLBB over LCNB, that is, the LCNB scenario would be more expensive and simultaneously result in an increase in breast cancer mortality. A related question is whether LCNB, being a less-invasive diagnostic procedure, would also be applied to women with nonpalpable breast lesions, who currently would have had no biopsy at all. The latter effect may lead to an increase in overall expenditures, yet, might result in an increasingly favourable cost-effectiveness of LCNB due to economies of scale (decrease of costs per procedure). In our study, however, we found that a lower threshold for breast biopsy resulted in a more favourable incremental cost-effectiveness ratio of NLBB compared to LCNB, even if low costs of LCNB were taken into account. Thus, from the payers’ perspective, a change in practice in terms of lower thresholds for referral due to the introduction of LCNB would result in an increase in costs without significant health effects. It would appear that, with the advent of LCNB, strict guidelines for referral should be continued.

A trend towards shorter hospitalisation or towards the outpatient setting for NLBB, which is common in other countries, will result in lower costs of NLBB procedures. Also, this would reduce the incremental cost-effectiveness ratio of NLBB compared to LCNB.

We further assumed that, if LCNB became widespread, FNA cytology would not be undertaken. In our country, preoperative FNA cytology – requiring specific expertise and not giving definite pathologic confirmation of tumour type – is performed on a relatively small scale. The role of FNA cytology is likely to become less important after the introduction of LCNB, but whether actual practice will mirror this assumption needs to be seen.

The MISCAN model simulates the effect of replacing NLBB by LCNB in women with nonpalpable breast lesions detected at screening. The model was not built to simulate the health effects and costs of LCNB for nonpalpable lesions that are detected outside the screening programme. In the Netherlands, the number of nonpalpable lesions detected outside the context of the breast cancer-screening programme is even larger than the number detected at screening. Had we been able to take into account all nonpalpable lesions, implementation of LCNB would have resulted in a further decrease of costs and an increase in life-years lost, assuming that the test characteristics of LCNB are similar for nonpalpable lesions detected within and outside the breast cancer-screening programme.

In the Netherlands, the breast cancer incidence is about 128 per 100 000 women (all ages). This is comparable to the incidence in the United Kingdom (about 136 per 100 000 women). There is no ground to assume that comparable test characteristics of LCNB cannot be achieved in other Western medical settings too. Finally, introduction of LCNB replacing NLBB for the diagnosis of nonpalpable lesions on a large scale, particularly in countries where breast screening results in an increased number of biopsies, will have a marked impact on health care costs.

## Figures and Tables

**Figure A1:**
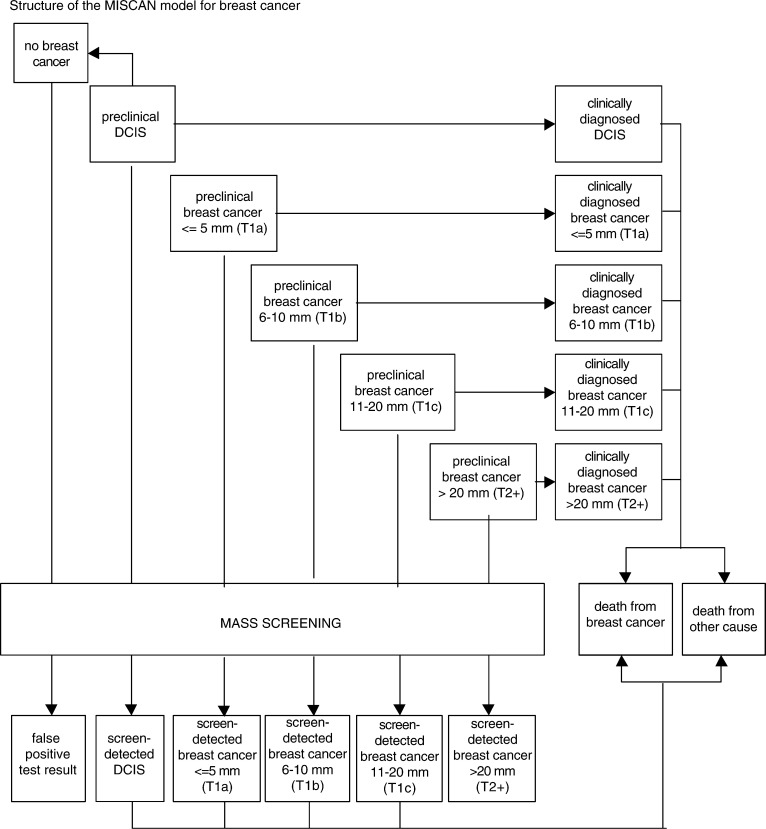
Structure of the MISCAN model for breast cancer.

## Appendix

### MIcrosimulation SCreening ANalysis (MISCAN) model

The core of the model consists of individual life histories, which are generated as a Markov process of stages and transitions in the natural history of breast cancer. The natural history is modelled as the onset of breast cancer (i.e. the transition from no disease to ductal carcinoma *in situ* (DCIS) or to a preclinical invasive T1a status), the transition through four possible preclinical invasive stages with increasing tumour diameters (T1a: tumour 0.5 cm or less in greatest dimension; T1b: tumour more than 0.5 cm but not more than 1 cm in greatest dimension; T1c: tumour more than 1 cm but not more than 2 cm in greatest dimension; and T2+: including the stages T2 (tumour more than 2 cm but not more than 5 cm in greatest dimension), T3 (tumour more than 5 cm in greatest dimension) and T4 (tumour of any size with direct extension to chest wall or skin)), and the transition from one of the preclinical stages into a clinically diagnosed stage (DCIS, T1a, T1b, T1c or T2+) until the patient dies from breast cancer or from other causes (see Figure A1). Transitions between stages depend on transition probabilities (see Table A1

**Table A1:** Important model parameters on natural history and screening in the MISCAN breast cancer model

*Mean duration (years) of screen-detectable preclinical stage by age*					
Stage	40	50	60	70	
Preclinical DCIS	5.2	5.2	5.2	5.2	
Preclinical T1a	0.1	0.1	0.1	0.2	
Preclinical T1b	0.4	0.5	0.7	0.9	
Preclinical T1c	0.8	1.0	1.5	1.8	
Preclinical T2+	0.6	0.8	1.1	1.4	
					
*Long-term survival by clinical stage and age*					
Age	DCIS	T1a	T1b	T1c	T2+
40	1.000	0.857	0.787	0.628	0.417
50	1.000	0.855	0.785	0.626	0.412
60	1.000	0.831	0.748	0.562	0.312
70	1.000	0.851	0.777	0.612	0.391
					
*Probability of surviving by time since diagnosis and stage*					
Time since diagnosis	T1a	T1b	T1c	T2+	
1 year	0.935	0.951	0.953	0.907	
3 years	0.745	0.854	0.838	0.698	
5 years	0.601	0.627	0.614	0.521	
7 years	0.497	0.481	0.437	0.399	
10 years	0.386	0.295	0.205	0.304	
20 years	0.201	0.182	0.145	0.132	
30 years	0.124	0.108	0.081	0.072	
50 years	0.000	0.000	0.000	0.000	
					
*Sensitivity of mammography by stage and age*					
Stage	Age 40–44 years	Age 45–49 years	Age ⩾50 years		
Preclinical DCIS	24%	32%	40%		
Preclinical T1a	39%	52%	65%		
Preclinical T1b	48%	64%	80%		
Preclinical T1c	54%	72%	90%		
Preclinical T2+	57%	76%	95%		
					
*Reduction in risk of dying of breast cancer by stage in which (pre)cancer is detected*					
Stage	Reduction in risk				
Preclinical DCIS	100%				
Preclinical T1a	89.2%				
Preclinical T1b	81.4%				
Preclinical T1c	56.7%				
Preclinical T2+	39.5%				
					
Health stage	Duration (de Haes *et al*, 1991)		Utility (de Haes *et al*, 1991)		
Terminal illness	1 month		0.712		
Palliative therapy+chemotherapy	4 months		0.469		
Palliative therapy+radiotherapy	1 month		0.419		
Palliative therapy+surgical therapy	5 weeks		0.383		
Palliative therapy+hormonal therapy	14 months		0.337		
Initial chemotherapy	6 months		0.283		
Initial radiotherapy	2 months		0.197		
Initial surgery	2 months		0.133		
Initial hormonal therapy	2 years		0.180		
Disease-free 2 months–1 year after mastectomy	10 months		0.156		
Disease-free 2 months–1 year after BCT	10 months		0.086		
Disease-free >1year after mastectomy	1 year		0.053		
Disease-free >1 year after BCT	1 year		0.040		
Screening attendance	1 week		0.006		

) and dwelling time distributions. Durations are generated from exponential distributions with stage- and age-dependent mean. Breast cancer screening is introduced as an intervention in the natural history, possibly resulting in the detection of breast cancer in earlier (preclinical) stages. Early detection allows early diagnosis and treatment and results in improved prognosis, that is, a reduction of breast cancer-specific morbidity and mortality.

Key parameters in the model are the mean duration of the preclinical screen-detectable stages, the sensitivity of the screening test and the improvement in prognosis after screen detection. The latter is defined as 1 minus the ratio of the risk of dying of screen-detected breast cancer divided by the risk if the same cancer had been diagnosed in the absence of screening. Age-specific assumptions with regard to the duration of the screen-detectable preclinical stages of breast cancer and the sensitivity of screening have been validated using the results of the Dutch breast cancer-screening programme (van den Akker-van Marle *et al*, 1999). The improvement in prognosis is based on the results of Swedish randomised trials (de Koning *et al*, 1995). Other model parameters describe particular screening characteristics (screening ages, interval and attendance rate).

The output of the model yields a number of estimates of the effect of screening and subsequent outcome. The numbers of women invited and screened are assessed, as well as the resulting number of true-positive and false-positive test results, and the number of cancers diagnosed outside the screening programme. The output also includes the numbers of diagnostic procedures for breast tumours detected at screening and for breast tumours detected outside the context of the breast cancer-screening programme, and the number of breast cancer therapies. Moreover, the model calculates the number of breast cancer deaths and life-years lost due to cancer.
